# Crystallization and preliminary crystallographic analysis of the bacterial capsule assembly-regulating tyrosine phosphatases Wzb of *Escherichia coli* and Cps4B of *Streptococcus pneumoniae*

**DOI:** 10.1107/S1744309109023914

**Published:** 2009-07-25

**Authors:** Hexian Huang, Gregor Hagelueken, Chris Whitfield, James H. Naismith

**Affiliations:** a Centre for Biomolecular Sciences, The University of St Andrews, Fife KY16 9RH, Scotland; b Department of Molecular and Cellular Biology, University of Guelph, Ontario N1G 2W1, Canada

**Keywords:** Wzb, Cps4B, kinases, phosphatases

## Abstract

Bacterial tyrosine kinases and their cognate phosphatases are key players in the regulation of capsule assembly and thus are important virulence determinants of these bacteria. Examples of the kinase/phosphatase pairing are found in Gram-negative bacteria such as *Escherichia coli* (Wzc and Wzb) and in Gram-positive bacteria such as *Streptococcus pneumoniae* (CpsCD and CpsB). Although Wzb and Cps4B are both predicted to dephosphorylate the C-terminal tyrosine cluster of their cognate tyrosine kinase, they appear on the basis of protein sequence to belong to quite different enzyme classes. Recombinant purified proteins Cps4B of *S. pneumoniae* TIGR4 and Wzb of *E. coli* K-30 have been crystallized. Wzb crystals belonged to space-group family *P*3_*x*_21 and diffracted to 2.7 Å resolution. Crystal form I of Cps4B belonged to space-group family *P*4_*x*_2_1_2 and diffracted to 2.8 Å resolution; crystal form II belonged to space group *P*2_1_2_1_2_1_ and diffracted to 1.9 Å resolution.

## Abstract 2

The crystallization is reported of two bacterial tyrosine phosphatases which belong to different enzyme families despite their ability to catalyse identical reactions.

## 1. Introduction

Gram-negative and Gram-positive pathogens often utilize capsules to evade and resist the immune system (Whitfield, 2006 ▶). Capsules are formed from carbohydrate polymers which are known as capsular polysaccharides (CPS) and form a discrete layer that covers the cell surface. Although there are several CPS biosynthesis systems, arguably the most widespread is the well studied Wzy carbohydrate polymerase-dependent pathway found in many Gram-positive and Gram-negative bacteria (Whitfield, 2006 ▶; Grangeasse *et al.*, 2007 ▶; Cuthbertson *et al.*, 2009 ▶). In the Wzy-dependent system, there is a requirement for both a tyrosine autokinase and a cognate phosphatase, and the available evidence suggests that the cycling of the phosphorylation state is crucial to the synthesis and export of CPS. Wzc from *Escherichia coli* and CpsCD *Streptococcus pneumoniae* are members of the tyrosine BY kinase family and are dephosphorylated by the phosphatases Wzb and CpsB, respectively (Grangeasse *et al.*, 2007 ▶).

Although the two phosphatases essentially catalyse the same reaction, Wzb and CpsB share no meaningful sequence identity and in fact belong to separate enzyme families. The NMR structure of Wzb (Lescop *et al.*, 2006 ▶) confirmed that it belongs to the low-molecular-weight protein tyrosine phosphatases (LMW-PTPs), which occur in both prokaryotes and eukaryotes (Grangeasse *et al.*, 2007 ▶). LMW-PTPs use a conserved cysteine nucleophile that generates an enzyme-bound thiophosphate ester intermediate, thereby liberating the tyrosine. The intermediate is then resolved in a second step by a water molecule (Ramponi & Stefani, 1997 ▶). CpsB phosphatases belong to the polymerase and histidinol phosphatase (PHP) family (Aravind & Koonin, 1998 ▶) and unlike Wzb they appear to be metal-dependent (Morona *et al.*, 2002 ▶; Mijakovic *et al.*, 2005 ▶; LaPointe *et al.*, 2008 ▶). No structural information for CpsB-like phosphatases is currently available. We wish to understand how these clearly different enzymes catalyse the same reaction and in doing so how they recognize the substrate. Capsule assembly is a potential therapeutic target and as the phosphatases are essential to this, a structural understanding of the phosphatases may offer a basis for rational drug design.

## 2. Expression and purification

The *cps4b* gene from *S. pneumoniae* TIGR4 was amplified from genomic DNA by PCR using the following oligonucleotides: cps4bfwd, 5′-GGC GGC CCA TGG GCA TGA TAG ACA TCC ATT CGC ATA TCG-3′, and cps4brev, 5′-GCC GCC GTC GAC CTA AAT TAG TTG ATC CAT TAC AAT TTT TCG-3′. The PCR product was cloned into the pEHISTEV vector (Liu & Naismith, 2009 ▶). Plasmid pWQ145 contains the *wzb* gene cloned in pET28a(+) and details of the construct have been described elsewhere (Wugeditsch *et al.*, 2001 ▶). Both constructs have an N-terminal His tag and the *cps4b* construct contains an additional tobacco etch virus (TEV) cleavage site between the His tag and the protein-coding sequence. An identical expression and purification procedure was followed for both proteins. The plasmids were transformed into *E. coli* Rosetta cells. A single colony was selected and grown overnight in Luria broth (LB) medium containing 50 µg ml^−1^ kanamycin and 37 µg ml^−1^ chloramphenicol. The overnight culture was used to inoculate 5 l LB medium at a ratio of 1:100. This culture was then grown at 310 K with shaking at 200 rev min^−1^. Once the culture had reached an optical density (600 nm) of 0.4, the incubation temperature was lowered to 288 K. At an OD_600_ of ∼1.0, protein expression was induced by adding isopropyl β-d-1-thiogalactopyranoside (IPTG) to a final concentration of 1 m*M* and the culture was incubated at 288 K overnight. Cells were harvested by centrifugation at 7000*g* for 30 min. Cell pellets containing the target proteins were resuspended in lysis buffer (20 m*M* phosphate pH 7.0) containing Benzonase (20 µg ml^−1^; Sigma) and Complete protease-inhibitor cocktail tablets (Roche Diagnostics; one tablet per 50 ml extract) and the mixture was stirred at 277 K for 30 min. A cell disrupter (207 MPa; Constant Cell Disruption Systems, Daventry, England) was used to disrupt the cells and the lysate was clarified by centrifugation at 30 000*g* for 1 h at 277 K. The cell-free supernatant was incubated with Ni–NTA resin (Sigma HIS-Select HF Nickel Affinity Gel) for 1 h at 277 K. Prior to loading, the resin was equilibrated in lysis buffer containing 100 m*M* NaCl. The loaded resin was washed with buffer *A* (50 m*M* Tris–HCl pH8, 100 m*M* NaCl) containing 50 m*M* imidazole and the target proteins were eluted with buffer *A* containing 500 m*M* imidazole. For Wzb, 5 m*M* dithiothreitol (DTT, final concentration) was added to the eluted protein. The Cps4B protein was incubated with 5 mg TEV protease overnight at 277 K. The protease and uncleaved protein were removed by a second metal-affinity column. For final purification, the proteins were passed over a Superdex 200 16/60 column (GE Healthcare) and eluted with buffer *A* (containing 5 m*M* DTT for Wzb). Fractions with appropriate purity as judged by SDS–PAGE were pooled and concentrated to 10 mg ml^−1^ for crystallization. Protein identity and integrity were confirmed by mass spectrometry. The proteins were flash-frozen in liquid N_2_ using thin-walled PCR tubes and stored at 193 K prior to further use.

## 3. Crystallization

Initial crystallization trials for Wzb and Cps4B were performed using a Honeybee 963 robot system (Genomic Solutions) and the following commercially available crystallization screens: JCSG+ and pHClear from Nextal, Classics and PEG/Ion from Hampton Research and Cryo II and Wizard from Emerald BioSystems. For each of the 96-­well sitting-drop vapour-diffusion screens (MRC plates, Wilden), 150 nl protein solution was mixed with 150 nl precipitant and equilibrated against a reservoir of 75 µl precipitant. The sealed plates were then incubated at 293 K.

**Figure 1:**
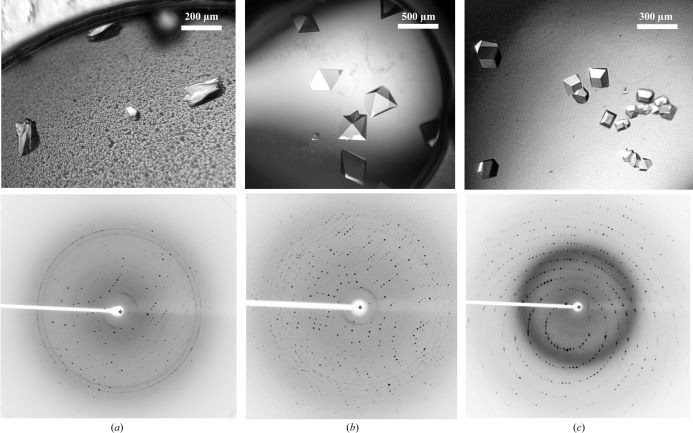
Crystals (upper panels) and the corresponding diffraction patterns (lower panels) of (*a*) Wzb (space group *P*3_*x*_21), (*b*) Cps4B crystal form I (space group *P*4_*x*_2_1_2) and (*c*) Cps4B crystal form II (space group *P*2_1_2_1_2_1_).

Initial Wzb crystals were obtained in condition B7 of the PEG/Ion Screen HT (Hampton Research) and were further optimized by several rounds of 24-well format hanging-drop experiments in which the precipitant concentration and pH were varied. The best results, as judged by crystal appearance, were obtained by mixing 1 µl protein solution (17 mg ml^−1^) with 1 µl well solution consisting of 0.2 *M* K_2_HPO_4_, 18%(*w*/*v*) PEG 3350, 0.1 *M* Tris–HCl pH 8.0. Crystals appeared after 1 d at 293 K (Fig. 1 ▶ *a*). Prior to flash-freezing in liquid nitrogen for data collection, the crystals were cryoprotected by transferring them to a cryosolution consisting of crystallization mother liquor supplemented with 15% glycerol.

Cps4B crystallized in two different crystal forms [crystal form I using condition No. 4 of Crystal Screen (Hampton Research); crystal form II using condition No. 38 of Cryo II (Emerald BioSystems); Figs. 1 ▶ *b* and 1 ▶ *c*]. Again, several rounds of hanging-drop optimization experiments were required to improve the initial crystallization conditions. Optimized crystals of form I were obtained by mixing 1 µl protein solution (10 mg ml^−1^) with 1 µl well solution (2 *M* ammonium sulfate, 0.1 *M* Tris–HCl pH 8.5). Crystals of form I appeared after 2 h at 293 K. The crystals were cryoprotected with crystallization mother liquor supplemented with 20% glycerol. Optimized crystals of form II were obtained by mixing 1 µl protein solution (10 mg ml^−1^) with 1 µl well solution [0.1 *M* sodium acetate, 35%(*w*/*v*) PEG 400 pH 4.2] and appeared after two weeks at 293 K. Cryoprotection was not necessary for crystal form II.

## 4. Preliminary crystallographic characterization

The Wzb crystals diffracted to 2.7 Å resolution. A single crystal was used to collect X-ray diffraction data over a range of 150° (0.5° steps) on the IO2 beamline (λ = 0.9796 Å) at the Diamond Light Source (Didcot, England), which is equipped with an ADSC Q315 detector. The data were indexed and processed in the trigonal space-group family *P*3_*x*_21 using *HKL*-2000 (Otwinowski & Minor, 1997 ▶). Data-collection statistics are shown in Table 1 ▶. Assuming a reasonable solvent content of 58%, the asymmetric unit of the Wzb crystals contains two Wzb monomers, corresponding to a Matthews coefficient of 2.94 Å^3^ Da^−1^. *MOLREP* from the *CCP*4 suite was used to calculate a self-rotation function (Collaborative Computational Project, Number, 1994 ▶). The χ = 180° section did not reveal any clear-cut noncrystallographic twofold axis. Thus, if the asymmetric unit indeed contains two Wzb molecules they are either not related by a twofold axis or the latter is parallel to one of the crystallographic twofold axes. In gel-filtration experiments Wzb elutes as a monomeric protein, indicating that the possible dimer in the asymmetric unit of the Wzb crystals is not functionally relevant. We expect to solve the structure of Wzb by molecular replacement using an NMR model of Wzb from *E. coli* K-12 (Lescop *et al.*, 2006 ▶). The K-30 and K-12 Wzb homologues share 51% identical amino acids.

Cps4B crystals of crystal form I belonged to the space-group family *P*4_*x*_2_1_2 and diffracted to 2.8 Å resolution. In contrast, crystals of form II belonged to the orthorhombic space group *P*2_1_2_1_2_1_ and diffracted to >1.9 Å resolution. In both cases a single crystal was used to collect X-ray diffraction data over a range of 180° in steps of 0.25° using Cu *K*α radiation on a Rigaku Saturn 944 detector. The data were indexed and processed using the program *XDS* (Kabsch, 1988 ▶). The data-collection statistics for both crystal forms are listed in Table 1 ▶. As judged by the calculation of Matthews coefficients (*P*4_*x*_2_1_2, 3.2 Å^3^ Da^−1^; *P*2_1_2_1_2_1_, 2.60 Å^3^ Da^−1^), both crystal forms contained a single molecule of Cps4B per asymmetric unit. Since the PDB database does not contain a suitable model that could be exploited to solve the Cps4B structure by molecular replacement, our efforts are now focused on gaining experimental phases for the Cps4B crystals.

## Figures and Tables

**Table 1:** Experimental X-ray data Values in parentheses are for the highest resolution shell.

		Cps4B	
	Wzb	Crystal form I	Crystal form II
Space group	*P*3_*x*_21	*P*4_*x*_2_1_2	*P*2_1_2_1_2_1_
Crystal system	Trigonal	Tetragonal	Orthorhombic
Unit-cell parameters (Å, °)	*a* = 90.0, *b* = 90.0, *c* = 83.5, α = β = 90, γ = 120	*a* = 89.5, *b* = 89.5, *c* = 91.7, α = β = γ = 90	*a* = 44.0, *b* = 58.5, *c* = 114.8, α = β = γ = 90
Matthews coefficient (Å^3^ Da^−1^)	2.94	3.21	2.60
Molecules per ASU	2	1	1
Resolution range (Å)	50.0–2.74	30.0–2.8	30.0–1.91
Mosaicity (°)	0.5	0.2	0.1
Total observations	92428	64805	202027
Unique reflections	10664	17410	40850
Completeness (%)	99.4 (100)	98.5 (85.1)	92.5 (61.4)
*R*_merge_ (%)	7.0 (45.5)	11.9 (46.4)	1.9 (4.4)
Multiplicity	8.7	3.7	5.0
*I*/σ(*I*)	27.3 (5.4)	14.3 (4.2)	64.4 (27.8)
